# Burn-out syndrome in healthcare practitioners- a narrative literature review

**DOI:** 10.1192/j.eurpsy.2021.702

**Published:** 2021-08-13

**Authors:** O. Vasiliu

**Affiliations:** Psychiatry, University Emergency Central Military Hospital Dr. Carol Davila, Bucharest, Romania

**Keywords:** burn-out, COVID-19, first line responders, healthcare practitioners

## Abstract

**Introduction:**

Burn-out syndrome is an underestimated entity in the medical environment, and lack of health policies for screening, prevention and early phase treatment strategies may be responsable for complications of this syndrome, e.g. major depression, substance use disorders, or anxiety disorders. Large variations in the estimation of burn-out prevalence in healthcare providers may be related to poorly designed epidemiological trials and lack of well-defined criteria for diagnosis.

**Objectives:**

To analyse the current evidence in the literature about the diagnosis and treatment of burn-out syndrome in physicians and auxiliary personnel.

**Methods:**

A literature review was performed through the main medical databases (Cochrane Database of Systematic reviews, PubMed, Thomson Reuters/Web of Science, SCOPUS, EMBASE, CINAHL) using the search paradigm “burn-out” AND “healthcare providers” OR “physicians” AND “nurses”. All papers published between January 2000 and August 2020 were included in the primary analysis.

**Results:**

A large number of papers were detected in the primary analysis (n=245), but only 15 remained after the inclusion/exclusion criteria were applied. Maslach Inventory for Burnout is the most extensively used instrument for screening, but its validity is questioned, and new instruments have been created, but less frequently applied. Cognitive behavioral therapy led to improvement of the emotional exhaustion in multiple trials. Meditation techniques, music therapy, structured physical exercises, and management-related interventions have been associated with low to moderate degree of success.

**Conclusions:**

Burn-out syndrome is a still insufficiently explored diagnosis and more good-quality epidemiological and clinical trials are needed in order to support adequate prevention and treatment strategies.

